# CORRIE: enzyme sequence annotation with confidence estimates

**DOI:** 10.1186/1471-2105-8-S4-S3

**Published:** 2007-05-22

**Authors:** Benjamin Audit, Emmanuel D Levy, Wally R Gilks, Leon Goldovsky, Christos A Ouzounis

**Affiliations:** 1 Laboratoire Joliot-Curie and Laboratoire de Physique, CNRS UMR5672, Ecole Normale Supérieure, 46 Allée d'Italie, F-69364 Lyon CEDEX 07, France; 2 Computational Genomics Group, The European Bioinformatics Institute, EMBL Cambridge Outstation, Cambridge CB10 1SD, UK; 3 Current address: Computational Genomics Group, MRC Laboratory of Molecular Biology, Hills Rd, Cambridge CB2 2QH, UK; 4 Medical Research Council Biostatistics Unit, Institute of Public Health, Cambridge CB2 2SR, UK; 5 Current address: Department of Statistics, School of Mathematics, University of Leeds, Leeds LS2 9JT, UK; 6 Current address: Computational Genomics Unit, Center for Research & Technology Hellas, PO Box 361, GR-57001 Thessalonica, Greece; 7 Current address: Institute of Agrobiotechnology, Center for Research & Technology Hellas, PO Box 361, GR-57001 Thessalonica, Greece

## Abstract

Using a previously developed automated method for enzyme annotation, we report the re-annotation of the ENZYME database and the analysis of local error rates per class. In control experiments, we demonstrate that the method is able to correctly re-annotate 91% of all Enzyme Classification (EC) classes with high coverage (755 out of 827). Only 44 enzyme classes are found to contain false positives, while the remaining 28 enzyme classes are not represented. We also show cases where the re-annotation procedure results in partial overlaps for those few enzyme classes where a certain inconsistency might appear between homologous proteins, mostly due to function specificity. Our results allow the interactive exploration of the EC hierarchy for known enzyme families as well as putative enzyme sequences that may need to be classified within the EC hierarchy. These aspects of our framework have been incorporated into a web-server, called CORRIE, which stands for Correspondence Indicator Estimation and allows the interactive prediction of a functional class for putative enzymes from sequence alone, supported by probabilistic measures in the context of the pre-calculated Correspondence Indicators of known enzymes with the functional classes of the EC hierarchy. The CORRIE server is available at: .

## Background

The explosion of genome sequencing technologies has resulted in an ever-increasing gap between the discovery of new gene sequences and their experimental characterization. The accumulation of raw sequence data has dictated the use of computational techniques for the inference of their possible functional roles, based on the evolutionary conservation of structure and function. However, this widely used empirical process has not attracted sufficient attention as a fundamental problem in computational biology, requiring rigorous analysis.

The typical solution to annotation transfer involves the inference of functional properties based on sequence similarity [1]. This procedure can be divided into two steps: (i) the establishment of a list of proteins of known function and significant sequence similarity to the uncharacterized sequence [2]; (ii) the selection of those characterized sequences from which the annotation might be transferred [3]. The procedure relies on the assumption of a strong relationship between protein structure and function. Despite the fact that this hypothesis is strongly supported by various studies [4], there is concern that a blind application of such procedures usually leads to annotation errors [5-8]. Two major types of errors can be made: (i) the short-listed homologous protein(s) have a different function from the query sequence (erroneous assignment, despite correct reference); (ii) the transferred annotations are incorrect (erroneous reference, despite correct assignment). The latter type followed by an iterative usage of annotation transfer results in the important problem of error propagation in annotated databases [3,9]. Modeling studies have demonstrated that dramatic consequences on the reliability of database annotations can thus arise, with detrimental effects for the quality and integrity of reference databases [9]. One of the challenges for future improvements is the association of function assignments with a measure of reliability that can control annotation quality [3], by excluding spurious annotations. Herein, we address this issue by analysing the Enzyme Classification (EC) hierarchy within a probabilistic framework for the process of homology-based annotation, as a follow-up of a previous theoretical study [10].

## Methods and results

Our approach relies on the usage of a reference dataset such as the EC hierarchy, where protein sequences are pre-classified into (an arbitrary number of) functional classes [10]. An assignment corresponds to a membership in a functional class; thus, function sharing becomes an explicit property. The possibility for a protein to belong to a functional class is assessed based on its similarity relationships with all protein sequences that do or do not belong to that class. Most existing methods map functions to proteins via the clustering of proteins based on sequence similarities irrespectively of any function sharing and the compilation of available functional descriptions in the (most relevant) cluster(s) to annotate the uncharacterized sequence(s) [11-13]. An innovative feature of our strategy is that individual sequences are mapped to functional classes, instead of individual functions mapped to sequence classes [10].

**Figure 1:**
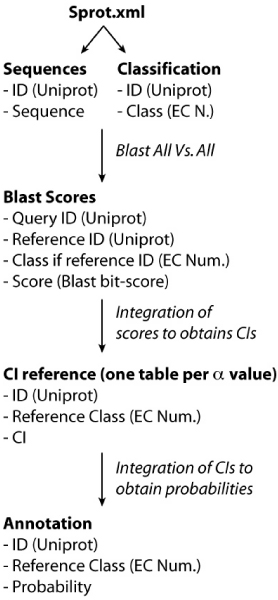
**Schematic view of the CORRIE annotation framework. **The only requirement for CORRIE is a classification of sequences. Here, we start with the classification of enzymes found in SwissProt. This enables us to create two tables, one for sequences and one for classes. From pairwise sequence comparisons we derive a score table, which describes all the classes hit by each sequence. BLAST scores are further integrated into correspondence indicators (CIs), which describe the relationship each sequence has with the classes it hits. Next, CIs are integrated to compute the probability that a sequence belongs to a particular class. The table "CI reference" is central to the framework as it constitutes a reference against which new proteins are compared and classified. This is illustrated in Figure 2.

We introduced Correspondence Indicators (CIs) as a novel measure to quantify the relationship between a protein sequence and a functional class. A CI results from the combination of pairwise similarity scores between a query sequence of interest and all the members of a functional class [10]. In our implementation, we use the BLAST bit-scores as a measure of pairwise similarity [14], but other measures can also be used (Figure 1). Herein, we provide an analysis of the ENZYME database [15], examine likely sources of error and announce the interactive server CORRIE.

**Figure 2:**
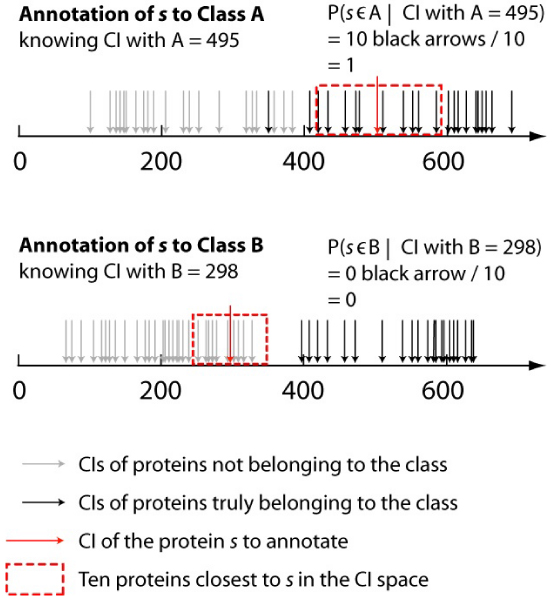
**Illustration of the probability calculation implemented in CORRIE**. To annotate a new sequence *s*, *s *is first aligned against all proteins in CORRIE. Here, *s *has similarity with proteins from two distinct classes: A and B. CIs between *s *and A, and between *s *and B are calculated [10]. The probability that *s *belongs to A (i.e. that *s *has function A) is calculated by comparison of the CI between *s *and A, with the CIs of proteins that belong or not to A. In this case, the ten proteins closest to *s *in the CI space are shown in the red dotted rectangle. Since all ten proteins truly belong to A, CORRIE estimates to P = 1 the probability for *s *to truly belong to A. When considering class B, ten proteins closest to *s *in the CI space do not belong to B. Therefore, CORRIE estimates to P = 0 the probability for *s *to truly belong to B. In this case, *s *would be annotated as having function A with probability 1.

The databases used in the present work were the ENZYME database (date:2006-07-12) [15] and UniProt/SwissProt (release 50.4, date:2006-07-25; UniProtKB 8.4) [16]. In total, we have obtained 77,812 proteins annotated as enzymes partitioned into 2,216 EC classes, of which 64,895 proteins partitioned into 827 classes were used: we have excluded enzymes with more than one EC number and all EC classes with ten or less members, as reported previously [10]. For sequence searches, we used BLAST (v.2.2.8) [14] with a bit-score cut-off threshold of 30. To filter low-complexity regions, we used CAST [17], with a threshold value of 25. The new interactive implementation of the annotation framework is implemented with MySQL (v.4.1) . All the results reported herein concern assignments (re-annotations) obtained with an assignment probability of one (P = 1) using the univariate method with α → ∞ i.e. with a CI Y_Ωj _reduced to the best BLAST hit of the query protein with class Ω_j _[10] (for an example, see Figure 2). As discussed previously, the univariate method has a lower coverage than the multivariate framework [10], yet since it treats the assignment to each class independently, it is more robust with respect to query proteins having more than one EC number assignment.

First, we followed the exact leave-one-out re-annotation scheme for assignments as described previously, with the updated information for proteins/EC classes [10], for comparison purposes. The overall (mean) performance was somewhat improved. We were able to generate (at P = 1) 59,766 assignments for 59,746 proteins (coverage 92.1%); some proteins may have more than one assignment at P = 1. Also, the number of annotation errors was 90, thus implying an error rate r = 0.15% (90 cases out of 59,766 assignments). Compared to our previous report [10], where we have annotated 28,088 enzymes over 589 classes, we observe an increase in coverage (92.1% compared to 90.6%) and a significant decrease in error rate (0.15% compared to 0.21%), despite a more than two-fold increase of the data.

Second, we have investigated in more depth the sources of error, by examining the local (specific) error rates. More precisely, we consider the probability that a re-annotation is an error knowing the annotation made by our approach, regardless of the true class, i.e. P(annotation is wrong | annotation by CORRIE). This analysis can only be performed at the P = 1 level because there is not enough information at P levels < 1 (due to the very high coverage of the database at P = 1). The results here are quite impressive: 799 (out of 827) classes have at least one assignment at level P = 1. For 755 of these classes, we did not observe any re-annotation error (again at P = 1). This corresponds to 51,131 out of 59,766 re-annotations, or a coverage level of 86%, with a specific error rate equal to zero. For the remaining 44 classes, there is at least one error recorded, which leads to non-zero specific error rates. These non-zero error rates vary across classes between 100% (1 error for 1 assignment) to 0.24% (4 errors for 1673 assignments). The highest error where the number of errors is more than one is 13.6% (3 errors for 22 assignments). We report all nine cases where the number of errors is more than one (Table 1). This information is also available on the web site, to help users assess annotation quality for specific classes in the EC hierarchy where the annotation process can be very challenging.

Third, we defined a distance measure in the re-annotation space in order to obtain a better understanding of the structure/function relationship for enzymes. This measure, denoted as δ (i → j) = N_ij_/N_i_, is the rate of re-annotation of proteins to class j, while they truly belong to class i; Ni is the number of proteins truly in class i, and Nj is the count of those assigned to class j. Note that this measure is not symmetric, i.e. δ (i → j) ≠ δ (j → i). For i = j, the δ measure provides a measure of recall, or in other words, it indicates whether there exists a high level of sequence specificity within class i. Typical example cases of low recall for two large families are for EC 1.10.2.2 (ubiquinol-cytochrome c reductase) [18], where δ = 13/89 (15%) and for EC 3.2.1.4 (cellulase) [19], where δ = 19/104 (18%). For i ≠ j, high values of the δ measure imply that errors are specifically made from class i to j (as opposed to errors randomly distributed over all classes). Hence, high values for δ (i → j) and δ (j → i) strongly suggest that merging the two classes would result in a much improved assignment of function based on sequence, or that those specific sequences do not contain enough information to distinguish the two enzymatic functions within the EC hierarchy. We report all six cases where the number of errors is more than two (Table 2), a surprisingly low number which demonstrates the high quality of assignments made by CORRIE in a series of control experiments.

Finally, we have implemented this strategy into a web-server called CORRIE implemented using MySQL and we announce its availability for wider use by the community. The software requires a reference set of protein sequences, their association to a functional classification and an all-vs-all similarity table. Then, for any unclassified query sequence, CORRIE generates a probability for its membership to a functional class. CORRIE has been made accessible at ; a downloadable version will follow soon. The format of the results is simple – by providing a query sequence, the user obtains the following information: the query sequence identifier, the original description (from the FASTA file format), an internal CORRIE protein identifier for retrieval purposes, the assignment probability, the predicted EC class, the EC description, and the local error rate for the specific class (as a guide for the quality of annotations) (Figure 1). The server provides all annotations obtained by CORRIE (including those with P < 1). The users may also use different α values and the multivariate framework. Users can also browse through various results so that they can refine their assessment of annotation quality and generally explore structure/function relationships within the entire sequence space of proteins known to be associated with enzymatic functions.

## Conclusion

We have previously developed a framework for the probabilistic annotation of enzymes into the functional classes of the EC hierarchy [10]. We have now extended this work using a larger reference database, and have reduced the error rates significantly while maintaining a coverage of >90%. We have also examined the local errors made in this assignment process and identified those EC classes more prone to non-specific structure/function relationships. Finally, we have made the system available as an interactive web server for the exploration of enzyme sequence space.

It is interesting to note that most errors reported (Tables 1 and 2) occur between closely related EC classes. This is particularly evident in cases where the similarity and difference of the function between overlapping classes is described (Table 2). In all six cases, the overall function remains the same while the difference lies in substrate specificity or the reaction mechanism. Recent studies have shown that substrate specificity in four of these twelve overlapping classes can be modulated with a small number of mutations. For instance, it has been reported recently that a RNA polymerase function was obtained from a DNA polymerase using *in vitro *compartmentalization, and a mutant with a single mutation was among the optimal mutants at synthesizing RNA [20]. Also, in the case of a transporting ATPase, the specificity of transport from H^+ ^to Li^+ ^was achieved by just four mutations [21].

Beyond the issue of functional specificity, there is also an aspect of biological reality in the problematic cases, in terms of overlapping enzyme properties. In other words, these classes might represent activities that co-exist in the same enzyme. In the previous example of the DNA polymerase, it has also been reported that a mutant with just five mutations maintained a DNA polymerase activity, demonstrating that both these activities co-exist [20]. Also, in the case of glucanases, co-existence of endo- and exo-activities has been observed in some enzymes [22]. Finally, with starch glucosyltransferases, CORRIE annotates ADP-glucose specific enzymes as being NDP-glucose specific, which is less accurate yet valid.

These examples illustrate the intricate nature of the sequence-function relationship found among those few cases that CORRIE fails to annotate correctly, and point to the limitation of using sequence similarity as a distance measure between enzymes. Therefore, we envisage implementing other methods in CORRIE in the near future. For example, the sequences within each class could be used to create one or more sequence profiles against which a new sequence could be aligned to produce an alternative CI measure, possibly focusing on key residues [23,24]. This would increase the sensitivity and specificity to a point where these ambiguous classes can be detected accurately.

One shortcoming of CORRIE, since it is based on the ENZYME database for validation purposes, is the implicit assumption that the query sequences are enzymes. A possible future development would be the explicit detection of enzyme sequences from similarity information. Schemes that have addressed the issue of enzyme recognition have been previously proposed [25]. This can be achieved by an all-vs-all comparison and further classification using CORRIE, with the entire UniProt database. In that setting, hypothetical proteins that would match known enzyme classes, could readily be assigned to specific EC numbers, with the proper probabilistic measures attached to them. Currently, this is possible, but the error rate is certainly under-estimated. Finally, the extension to other classification schemes (and semantically richer formats) will facilitate the assignment of protein sequences to various aspects of biological function beyond the EC hierarchy.

## Competing interests

The authors declare that they have no competing interests.

## Authors' contributions

BA, LG and CAO participated in the design and coordination of the study. BA, LG and EDL developed the software code and the web site. All authors have drafted the manuscript, and subsequently have read and approved the final manuscript.

## Figures and Tables

**Table 1:** Local error rate per EC class, for those cases where there is more than one error.

**EC**	**Errors**	**Assignments**	**Error %**	**Description**
3.2.1.4	3	22	13.64	Cellulase
3.2.1.8	3	29	10.34	Endo-1,4-beta-xylanase
2.4.1.21	4	99	4.04	Starch synthase
1.6.5.3	9	457	1.97	NADH dehydrogenase (ubiquinone)
2.7.11.1	14	819	1.71	Non-specific Ser/Thr protein kinase
1.1.1.37	2	208	0.96	Malate dehydrogenase
3.6.3.14	14	1904	0.74	H^+^-transporting two-sector ATPase
4.2.1.33	2	310	0.65	3-isopropylmalate dehydratase
2.7.7.6	4	1673	0.24	DNA-directed RNA polymerase

Column names: EC – EC number assignment by CORRIE; Errors – number of errors assigned to this class; Assignments – total number of assignments to this class; Error % – the local error rate; Description – the description of the corresponding EC reaction.

**Table 2:** Overlapping EC classes, for those cases where there are more than two errors from a true EC class to an assigned EC class.

**True EC**	**Name of true class**	**Assigned EC**	**Name of assigned class**	**Common activity**	**Difference**
2.7.7.7	DNA-directed DNA polymerase	2.7.7.6	DNA-directed RNA polymerase	DNA-dependent nucleotidyltransferase	Substrate: DNA or RNA
1.6.99.5	NADH dehydrogenase (quinone)	1.6.5.3	NADH dehydrogenase (ubiquinone)	NADH dehydrogenase	Electron acceptor: quinone or ubiquinone
3.2.1.91	Cellulose 1,4-beta-cellobiosidase	3.2.1.4	Cellulase	Hydrolysis of 1,4-beta-D-glucosidic linkages	Exo-hydrolysis or endo-hydrolysis
2.7.1.137	Phosphatidylinositol 3-kinase	2.7.11.1	Non-specific Ser/Thr protein kinase	Kinase	Substrate: PI3 or Ser/Thr
2.4.1.242	NDP-glucose – starch glucosyltransferase	2.4.1.21	Starch synthase	Starch glucosyltransferase	Substrate: NDP-glucose or ADP-glucose
3.6.3.15	Sodium-transporting two-sector ATPase	3.6.3.14	H^+^-transporting two-sector ATPase	Ion transporting two sector ATPase	Ion specificity: NA^+ ^or H^+^

Column names: True EC/Name – the real EC number/name; Assigned EC/Name – the assigned properties made by CORRIE; Common activity/Difference – similarities and differences of substrate specificity and mechanisms for the corresponding reaction pairs.
